# A capsule-associated gene of *Cryptococcus neoformans*, *CAP64*, is involved in pH homeostasis

**DOI:** 10.1099/mic.0.001029

**Published:** 2021-06-14

**Authors:** Yumi Imanishi-Shimizu, Yukina Kamogawa, Yukino Shimada, Kiminori Shimizu

**Affiliations:** ^1^​ Department of Bioscience, College of Science and Engineering, Kanto Gakuin University, 1-50-1 Mutsuura-higashi, Kanazawa-ku, Yokohama 236-8501, Japan; ^2^​ Department of Biological Science and Technology, Tokyo University of Science, Niijuku 6-3-1, Katsushika-ku, Tokyo 125-8585, Japan

**Keywords:** basidiomycetous yeast, *CAP64*, exocytosis, quinacrine accumulation, vesicle acidification

## Abstract

The *CAP64* gene is known to be involved in capsule formation in the basidiomycete yeast *Cryptococcus neoformans*. A null mutant of *CAP64*, Δ*cap64*, lacks a capsule around the cell wall and its acidic organelles are not stained with quinacrine. In order to clarify whether the Cap64 protein indeed maintains vacuole or vesicle acidification, so that the vesicle containing the capsule polysaccharide or DBB substrate are transported to the cell membrane side, the relationship between *CAP64* and intracellular transport genes and between *CAP64* and enzyme-secretion activity were analysed. Laccase activity was higher in the Δ*cap64* strain than in the wild-type strain, and the transcriptional levels of *SAV1* and *VPH1* were also higher in the Δ*cap64* strain than in the wild-type strain. The intracellular localization of the Cap64 protein was analysed by overexpressing an mCherry-tagged Cap64 and observing its fluorescence. The Cap64 protein was accumulated within cells in a patch-like manner. The quinacrine-stained cells were observed to analyse the acidified cell compartments; quinacrine was found to be accumulated in a patch-like manner, with the patches overlapping the fluorescence of CAP64-mCherry fusion protein. Quinacrine was thus accumulated in a patch-like fashion in the cells, and the mCherry-tagged Cap64 protein position was consistent with the position of quinacrine accumulation in cells. These results suggest that *CAP64* might be involved in intracellular acidification and vesicle secretion via exocytosis.

## Introduction

Diazonium Blue B (DBB), 3,3′-dimethoxy-[1,1′-biphenyl]−4,4′-bis tetrachlorozincate, has been used to distinguish basidiomycetous yeasts from ascomycetes yeasts [1, 2]. Ascomycetous yeast colonies are not stained by DBB, but basidiomycetous ones are stained to red or purple. However, the molecular targets for DBB staining are still unknown. Identifying the targets would provide clues as to how their ancestors have evolved into the present basidiomycetes or ascomycetes. *Cryptococcus neoformans* is a model basidiomycetous yeast for molecular biological study, and was used to clarify DBB staining mechanisms in this study.

During our study of the mechanism of DBB staining, we found that only the cell edge of the *CAP64* mutant (Δ*cap64*) strain was not fluorescent when we performed a DBB staining test using *CAP* gene-deletion mutants [3]. We also determined that the *PMT2* gene coding protein *o*-mannosyl transferase is necessary for the DBB staining in *C. neoformans* [4]. The *PMT2* deletion mutant is negative by the DBB colony staining test but has a thick capsule around the cell wall [4]. These results suggested that the molecular targets for the DBB staining reaction might not be related to the capsule formations, and *CAP64* might play an additional role in the DBB staining.

Quinacrine diffuses across membranes and accumulates in intracellular acidic compartments, and it has therefore been used as a marker for yeast vacuoles and investigations into the morphology of yeast vacuoles or vesicles [5–7]. We investigated the characteristics and phenotype of the Δ*cap64* strain, and found that intracellular quinacrine accumulation was not observed in Δ*cap64*, but was clearly seen in the wild-type strain [3]. This result suggests that *CAP64* regulates the acidification of vacuoles or vesicles. Acidification of vesicular or vacuolar compartments plays an important role in many intracellular processes, such as protein secretion, glycosylation of proteins in the Golgi, osmotic and pH stability, and autophagic degradation [7–9]. A common vacuolar proton pump has been identified in Golgi-derived and vacuolar membranes in *Saccharomyces* yeast [9]. This vacuolar (H^+^)-ATPase proton pump contains about 13 subunits, including a regulatory subunit that is encoded by the *VPH1* gene [10]. In *C. neoformans*, the *VPH1* gene was isolated by screening for mutants defective in laccase activity, and its deletion mutant showed defects in capsule formation and urease expression [8]. On the other hand, it was shown that post-translational modifications of virulence factor expression involving polysaccharides depend on vesicular acidification [11].

The capsule of *C. neoformans* consists mainly of glucuronoxylomannan (GXM) which comprises more than 90 % of the capsule’s polysaccharide mass, and galactoxylomannan (GalXM) [12]. The polysaccharide composes an α−1, 3-linked mannose backbone which is *o*-acetylated, and substituted with glucuronic acid and xylose residues [13]. GXM synthesis is thought to occur in Golgi-related structures. GXM traffic to the cell surface involves transport by secretion of polysaccharide-containing vesicles, which cross the cell wall, releasing their contents into the extracellular space. The released polysaccharide is then connected to the cell wall or incorporated into the growing capsule [14].

In *Saccharomyces* yeast cells, one of the small GTPases regulating the exocytosis process is known as Sec4p [15]. In *C. neoformans*, a *SAV1-*encoding Sec4p homologue was cloned and this gene disruption resulted in a decrease in acid phosphatase secretion, and an accumulation of post-Golgi exocytotic vesicles in the cytoplasm [16]. The observation of these vesicles connecting with antibodies against GXM indicated that the GXM transport occurs via secretory vesicles derived from the Golgi apparatus.

In this study, in order to clarify the relation between *CAP64* and both *VPH1* and *SAV1*, the expression levels of *VPH1* and *SAV1* in the Δ*cap64* cells were investigated and were compared with the levels in wild-type. Then, the Cap64p localization pattern was also compared with the quinacrine staining pattern.

## Methods

### Strains and growth conditions

The strains used in this study are summarized in Table 1. Yeast cells were grown in potato dextrose agar (PDA) [24 g l^−1^ potato dextrose broth (Difco), 15 g l^−1^ agar] or YPDA (1 % yeast extract, 2 % polypeptone, 2 % glucose, 2 % agar) for a couple of days at 25 °C. For the induction of laccase production, asparagine medium [1 g l^−1^ l-asparagine, 0.5 g l^−1^ MgSO_4_・7H_2_O, 3 g l^−1^ KH_2_PO_4_, 1 g l^−1^ thiamine, 1 g l^−1^ biotin, 1 mM L−DOPA (Nacalai), pH5.6] was used [17]. *Escherichia coli* DH5α was the host strain for the recovery of a plasmid and was grown in LB medium [10 g l^−1^ polytone, 5 g l^−1^ bacto yeast extract (Difco), 10 g l^−1^ NaCl, pH 6.8] containing 25 µg ml^−1^ kanamycin for 12 h at 37 °C at 150 r.p.m.

**Table 1.** Strains used in this study

Strains	Genotype	Source
KN3501α	*MATα*	Nielsen *et al*. [33]
KN3501a	*MATa*	Nielsen *et al*. [33]
TYCC77	*MATα*, *cap64*::*ADE2*	Moyrand *et al*. [32]
KGU 10016	*MATα*, *cap64*Δ::*ADE2 CAP64*::*Hyg*	Imanishi *et al*. [3]
KGU 10048	*MATα*, *cap64*::*ADE2*, *CAP64*-mCherry::*Hyg*	This study
KGU 10049	*MATα*, *cap64*::*ADE2*, *CAP64*-mCherry::*Hyg*	This study
KGU 10050	*MATα*, *cap64*::*ADE2*, *CAP64*-mCherry::*Hyg*	This study
KGU 10051	*MATα*, *cap64*::*ADE2, CAP64*-mCherry::*Hyg*	This study
KGU 10052	*MATα*, *cap64*::*ADE2, CAP64*-mCherry::*Hyg*	This study
KGU 10053	*MATα*, *cap64*::*ADE2, CAP64*-mCherry::*Hyg*	This study
KGU 10054	*MATα*, *cap64*::*ADE2, CAP64*-mCherry::*Hyg*	This study
KGU 10055	*MATα*, *cap64*::*ADE2, CAP64*-mCherry::*Hyg*	This study
KGU 10056	*MATα*, *cap64*::*ADE2, CAP64*-mCherry::*Hyg*	This study
KGU 10057	*MATα*, *cap64*::*ADE2,CAP64*-mCherry::*Hyg*	This study
KGU 10058	*MATα*, *cap64*::*ADE2,* mCherry-*CAP64*::*Hyg*	This study
KGU 10059	*MATα*, *cap64*::*ADE2,* mCherry-*CAP64*::*Hyg*	This study
KGU 10060	*MATα*, *cap64*::*ADE2,* mCherry-*CAP64*::*Hyg*	This study
KGU 10061	*MATα*, *cap64*::*ADE2,* mCherry-*CAP64*::*Hyg*	This study

### Construction of an mCherry-tagging Cap64 overexpression strain

**Fig. 1.**
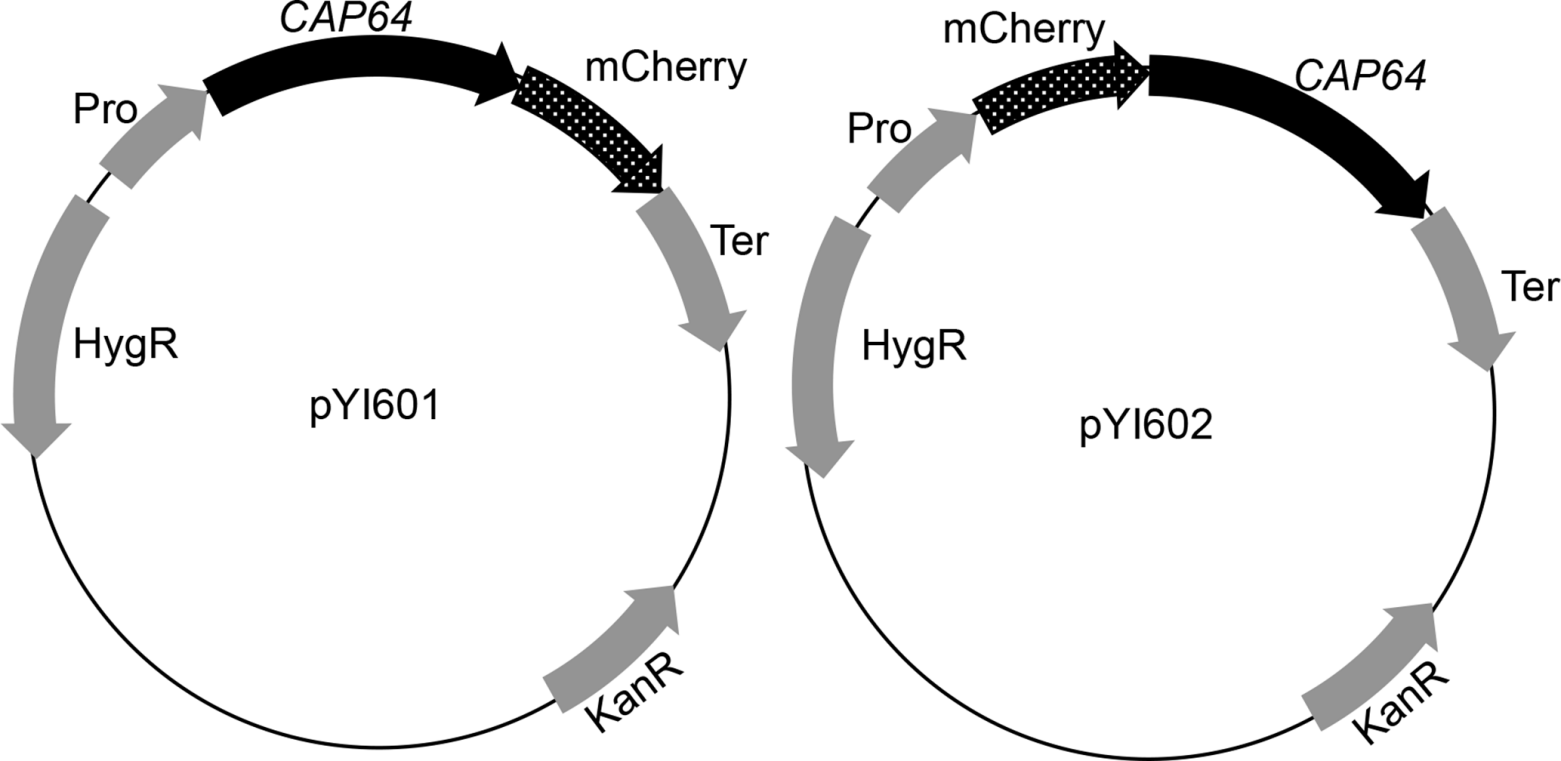
Two plasmids with an CAP64-mCherry cassette. Plasmid pYI601 has a cassette for expressing C-terminal mCherry-tagged Cap64p, and plasmid pYI602 has a cassette for expressing N-terminal mCherry-tagged Cap64p.

To study the intracellular localization of Cap64p in *C. neoformans* cells, a cassette coding for an mCherry-tagged Cap64p, designated the *CAP64*-mCherry cassette, was driven by the *GDP* promoter and was introduced into the Δ*cap64* strain. The cassette was constructed by overlap PCR using the oligos listed in Table 2 by the method described by Davidson *et al*. [18]. In order to insert an mCherry tag on the C-terminal side of Cap64p, an mCherry DNA fragment was PCR-amplified with the primers mcherry-F and mcherry-R using the plasmid pmCherry DNA as a PCR template. The vector containing the *CAP64* gene was PCR-amplified with the primers cap64-linker-F2 and cap64-linker-R2 using pKIS615. Those PCR products were combined and cloned using NEBuilder HiFi DNA Assembly Master Mix (New England Bio Labs), and pYI601 was constructed (Fig. 1). Its insert DNA was sequenced and confirmed to encode the designed protein. In order to insert an mCherry tag on the N-terminal side of Cap64p, a DNA fragment of *CAP64* was PCR-amplified with the primers cap64-mcherry and ter-cap64 using gDNA of the wild-type strain as template DNA, and the mCherry DNA fragment was PCR amplified with the primers link-mcherry-F and link-mcherry-R using the plasmid pmCherry DNA as a PCR template. The vector was PCR-amplified with the primers CAP64-vector and mche-vector using pKIS612 (Shimizu, unpublished) DNA digested by the restriction enzyme *Hin*dIII. These PCR products were combined and cloned into pKIS612 using NEBuilder HiFi DNA Assembly Master Mix (New England Biolabs), and pYI602 was constructed (Fig. 1). Its insert DNA was sequenced and confirmed to encode the designed protein. A plasmid carrying the *CAP64*-mCherry cassette was used as a template, and the DNA fragment of the cassette was amplified by PCR with the M13 forward primer and M13 reverse primer. The PCR product was purified using an EZ-10 Spin Column PCR Products Purification Kit (Bio Basic, Ontario, Canada) and introduced into cryptococcal cells as described previously [19].

**Table 2.** Primers used for PCR in this study

Primer name	Sequence (5′−3′)
*CAP64* primers for the qRT-PCR analysis	
CAP64_F	CTGATCAAACCGATCTCGTCATTCT
CAP64_R	GATCAGGTCTCACAAGGATGCTTCT
*LAC1* primers for the qRT-PCR analysis	
RT-lac1.f2	AACGGTACTCTGCAATTATCAACA
RT-lac1.r2	AACCATTCCATTACCCGTATACCT
*SAV1* primers for the qRT-PCR analysis	
SAV1_RT_F	ATCTGTTACGATTGAGCAAGGA
SAV1_RT_R	AATCGCTTTGCTTGTTGACATT
*VPH* primers for the qRT-PCR analysis	
VPH_RT_F	TGCCTCGATAAGTCCTGTAGAATT
VPH_RT_R	ATTACATTCCCATTCTTGTTTGCA
*ACT1* primers for the qRT-PCR analysis	
RT-ACT1.UP	ATGGAAGAAGAAGTCGCCGC
RT-ACT1.LP	TAGAAGGGAAGACAGCTCGG
mCherry primers for the qRT-PCR analysis	
mcherry_RT_R	CGAGTTCATCTACAAGGTGAAGCT
mcherry_RT_F	TACATGAACTGAGGGGACAGGA
Primers for construction of the cassette coding *CAP64* tagged with mCherry at the N-terminus of *CAP64* (pYI602)	
ter-cap64	TACTGTAACCCCATACTAGGTCCCAAAGCTACAGT
link-mecherry-R	GGCTTCTCTGAGCATCTTGTACAGCTCGTCCATGC
cap64-mcherry	GACGAGCTGTACAAGATGCTCAGAGAAGCCAAGGT
link-mecherry-F	TTAGCATAAATACAAATGGTGAGCAAGGGCGAGGA
CAP64-vector	AGCTTTGGGACCTAGTATGGGGTTACAGTAGCAGT
mche-vector	GCCCTTGCTCACCATTTGTATTTATGCtAAGTATA
Primers for construction of the cassette coding *CAP64* tagged with mCherry at the C-terminus of *CAP64* (pYI602) and for conforming transformants containing the cassette	
mcherry-F	TGTAGCTTTGGGACCATGGTGAGCAAGGGCGAGGA
mcherry-R	CTACTGTAACCCCATTTACTTGTACAGCTCGTCCA
cap64-linker-F2	GCCCTTGCTCACCATGGTCCCAAAGCTACAGTCGT
cap64-linker-R2	GAGCTGTACAAGTAAATGGGGTTACAGTAGCAGTA
pro-cap64	TTAGCATAAATACAAATGCTCAGAGAAGCCAAGGT
Primers for amplification of the cassette	
M13 forward primer	CGCCAGGGTTTTCCCAGTCACGAC
M13 reverse primer	AGCGGATAACAATTTCACACAGG

Genomic DNA was isolated from transformants and was confirmed to contain the DNA fragment of the CAP64-mCherry cassette by PCR amplification with the ter-cap64 and link-mcherry-R primers. The PCR product was detected by agarose gel electrophoresis and sequenced. For DNA extraction, yeast cells were harvested from PDA cultures for DNA extraction according to the benzyl chloride method described by Zhu *et al*., with minor modifications [20]. PCR was performed using the KAPA Taq EXtra HS ReadyMix PCR Kit (Nippon Genetics, Tokyo) according to the manufacturer’s instructions. PCR products were purified using the FastGene Gel/PCR Extraction Kit (Nippon Genetics) according to the manufacturer’s manual and directly sequenced on an ABI 3130xl genetic analyser (Applied Biosystems, Foster City, CA, USA). Sequences were analysed using GENETYX ver.11 software (Genetyx).

### Fluorescence microscopy

Quinacrine staining of vacuoles or vesicles was performed by the method described by Corbacho *et al.* (2012) with modifications [21]. Cells were collected by centrifugation and 5×10^6^ cells were resuspended in 1 ml of YPD buffer containing 50 mM phosphate (pH 7.6) and 100 mM quinacrine dihydrochloride hydrate (Tokyo Chemical Industry Co.). Cells were incubated for 15 min at 25 °C, and harvested cells were resuspended in 100 µl of 50 mM phosphate buffer (pH 7.6) and examined with a Leica optical microscope DM 2500 (Leica Microsystems, Wetzlar Germany) equipped with a fluorescence source (450/490 nm excitation, 510 nm dichroic mirror, 515 nm-emission). In order to observe the intercellular Cap64p localization, the mCherry-tagged Cap64 overexpression strain was inoculated into YPD medium and cultured for 20 h at 25 °C, and then the cells were examined with a Leica optical microscope DM 2500 equipped with a fluorescence source (515/560 nm excitation, 580 nm dichroic mirror, 590 nm-emission). Data analyses were performed using ImageJ [22].

For DAPI staining, cells were grown in YPD liquid medium for 20 h and collected by centrifugation. Cells were washed with sterilized distilled water and incubated in 95 % ethanol on ice for 1 h. Fixed cells were collected by centrifugation and suspended in PBS. Cells were collected by centrifugation, suspended in PEMS (100 mM PIPES, 1 mM EGTA, 1 mM MgCl_2_, 1.2 M sorbitol）containing 1 µg ml^−1^ DAPI (Merck, Darmstadt, Germany) and incubated at 30 °C for 30 min. After the incubation, cells were examined with a Leica optical microscope DM 2500 (Leica Microsystems) equipped with a fluorescence source (360/40 nm excitation, 400 nm dichroic mirror, 470/40 nm emission).

### Capsule observation

Yeast strains were grown according to the methods described previously [3]. To observe the capsule, a drop of Indica ink was added to the cell suspension on the slide grass, and the samples were observed using a Leica optical microscope DM 2500 (Leica Microsystems).

### Laccase activity

Strains were grown for 12 h in YPD at 25 °C with shaking at 150 r.p.m. After cultivation, cells were collected by centrifugation, washed twice in 50 mM PBS (pH 7.0), counted by hemocytometry, and adjusted to achieve an inoculum of 1×10^7^ cells ml^−1^. Cells were then incubated in asparagine medium for 24 h at 30 °C. After incubation, the supernatants were obtained after removal of the cells via centrifugation. The amount of pigment produced in the supernatants was determined by a spectrophotometer (U-3310; Hitachi, Tokyo) at a 475 nm wavelength. Assays were repeated three times.

### qRT-PCR

Total RNA extraction was performed using Trizol reagent (Life Technologies, Carlsbad, CA) and cDNA was synthesized using ReverTra Ace qPCR RT Master Mix with gDNA Remover (Toyobo, Osaka, Japan) following the provided instructions. The cDNA was used as a template in the qRT-PCR analysis using THUNDERBIRD qPCR MixSYBR (Toyobo) according to the manufacturer’s recommendations. Step One Plus Real-Time PCR (Thermo Fisher Scientific) was used to detect and quantify the PCR products. The PCR was conducted using the following protocol: incubation at 95 °C for 10 min followed by 40 cycles of 95 °C for 15 s and 60 °C for 1 min. Each set of PCR included a triplicate of each target gene. The data were normalized to the expression levels of the housekeeping gene *ACT1* (encoding actin) in each set of PCRs. The sequences of the primers for the qRT-PCR analysis are listed in Table 2.

## Results

### Deletion of *CAP64* inhibits the separation of daughter cells from mother cells

**Fig. 2.**
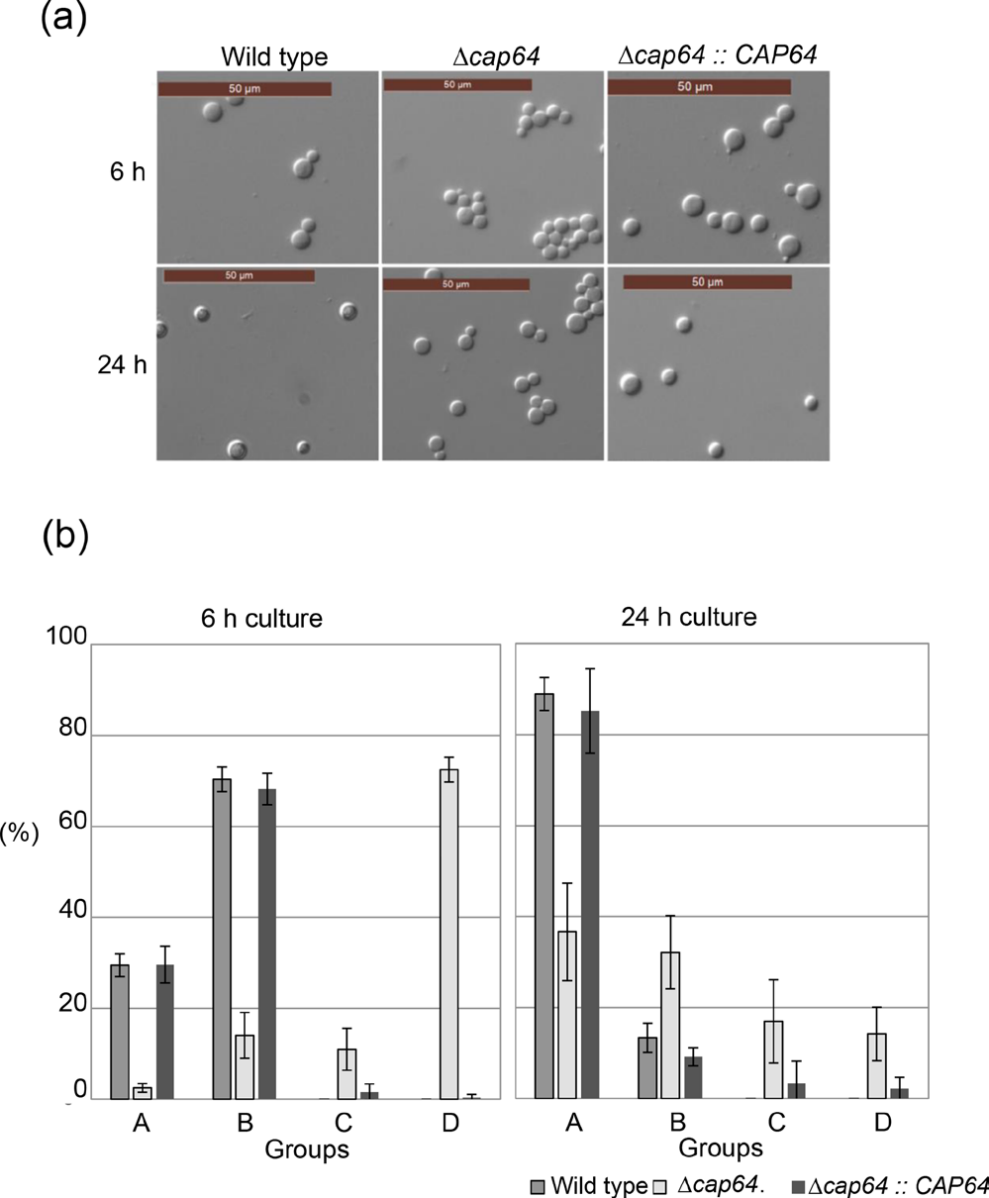
Cell morphology comparison among the Δ*cap64* strain, the wild-type strain KN3501α, and the *CAP64* complement strain KGU 10016. Cells were grown in YPD medium for 6 or 24 h and observed microscopically (**a**). Cells were classified into a single cell group (**a**), a two-cell connected group (**b**), a three-cell connected group (**c**), and a group of four or more connected cells (**d**). The number of cells in each group was counted and the ratio to the total number of cells observed in each culture was expressed as a percentage (**b**).

**Fig. 3.**
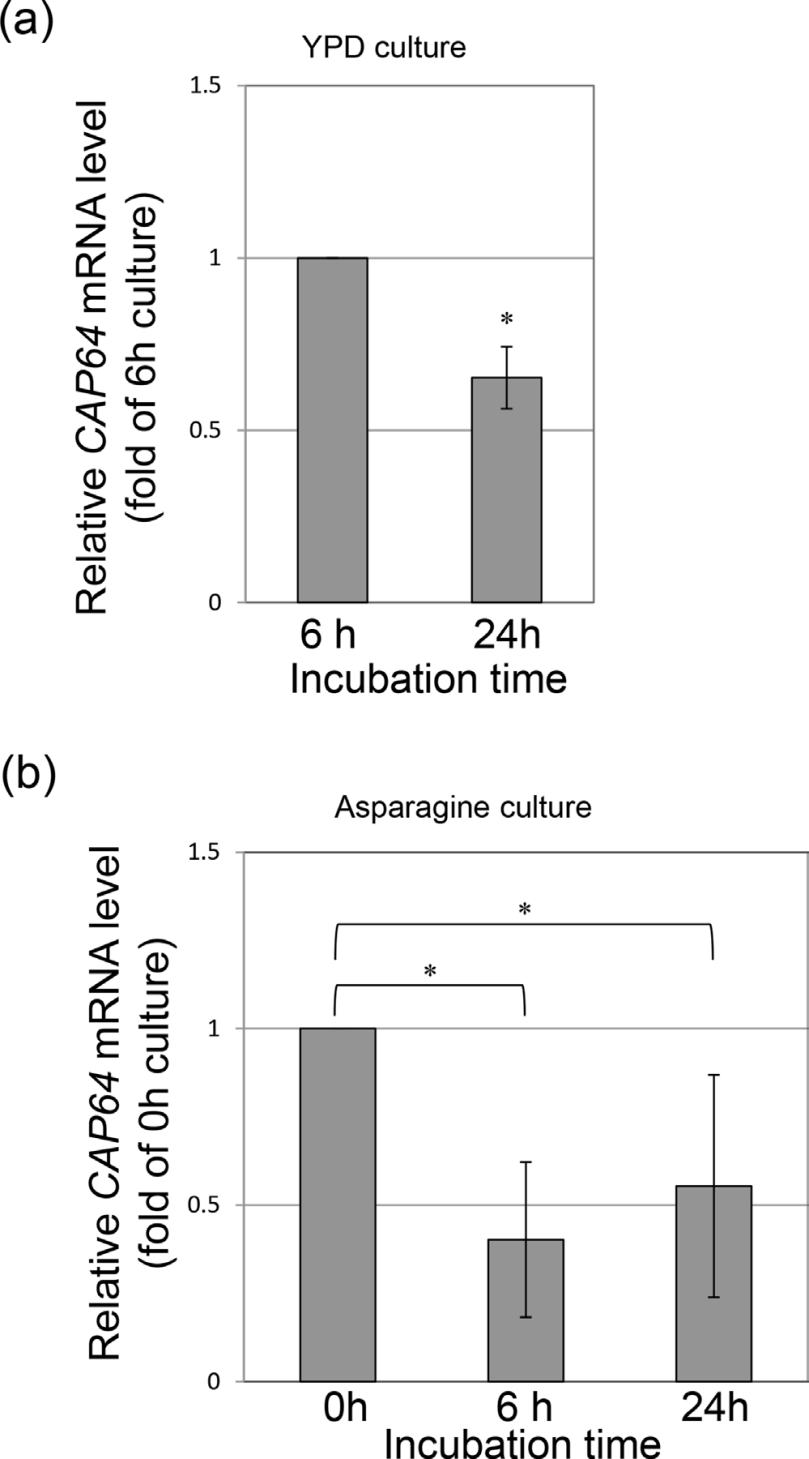
The amount of *CAP64* transcript in the wild-type strain culture was compared during growth in YPD medium. The amount of *CAP64* transcript was examined after culturing in YPD medium for 6 or for 24 h (**a**), and the amount of *CAP64* transcript was examined after culturing in asparagine medium for 6 or 24 h (**b**). Student’s *t*-test was used to compare them. Experiments were carried out in triplicate. Values are reported as the mean±SD. *Statistically significant by Student’s *t*-test (*P*<0.05).

The *CAP64* gene has been shown to be involved in capsule formation in *C. neoformans*, but its deletion mutant was negative for DBB staining, with irregular vacuole morphology [3]. *CAP64* thus seems to play an additional role other than capsule formation, and we therefore re-observed the cell morphology in detail. At first, the cell morphology of the Δ*cap64* strain was compared to that of the wild-type or *CAP64* complement strain. Cells were grown in a YPD liquid medium, and the cell morphology of the log phase (cells grown for 6 h) or of the stationary phase (cells grown for 24 h) was observed under a phase contrast microscope. The cell morphology of the Δ*cap64* strain at log phase revealed that several cells were connected and many daughter cells could not separate from their mother cells, although the cell morphology of the wild-type strain showed that only a few cells were connected and the cell division proceeded normally (Fig. 2a). For the purpose of statistical analysis, the cells were classified into four groups: a single cell group (A), a two-cell connected group (B), a three-cell connected group (C), and a group of four or more connected cells (D). The number of cells in each group was counted and the ratio to the total number of cells observed in each culture was expressed as a percentage (Fig. 2b). In the Δ*cap64* strain culture, 72.5 % of total cells were in group D at the log phase, and this ratio decreased to 12.2 % at the stationary phase. The ratio of the number of cells in group A increased from 1.6 % at the log phase to 36.6 % at the stationary phase. On the other hand, in the wild-type or the *CAP64* complement strain culture, almost all cells in these growth phases were classified into group A or group B. This result suggests that the gene deletion of *CAP64* inhibits the separation of daughter cells in the log phase, but then the inhibition is only partially released at the stationary phase. When the wild-type strain was grown in a YPD liquid medium, the amount of *CAP64* mRNA was analysed by qRT-PCR analysis. The amount of *CAP64* transcript at the stationary phase was 0.65-fold the amount in the log phase (Fig. 3a). The results of *CAP64* transcript analysis and the observation of the separation of daughter cells from mother cells indicated that gene deletion of *CAP64* inhibits the separation of daughter cells in log phase. But when growth enters the stationary phase, other factors might be related to cell separation.

### Correlation between *CAP64* and laccase

**Fig. 4.**
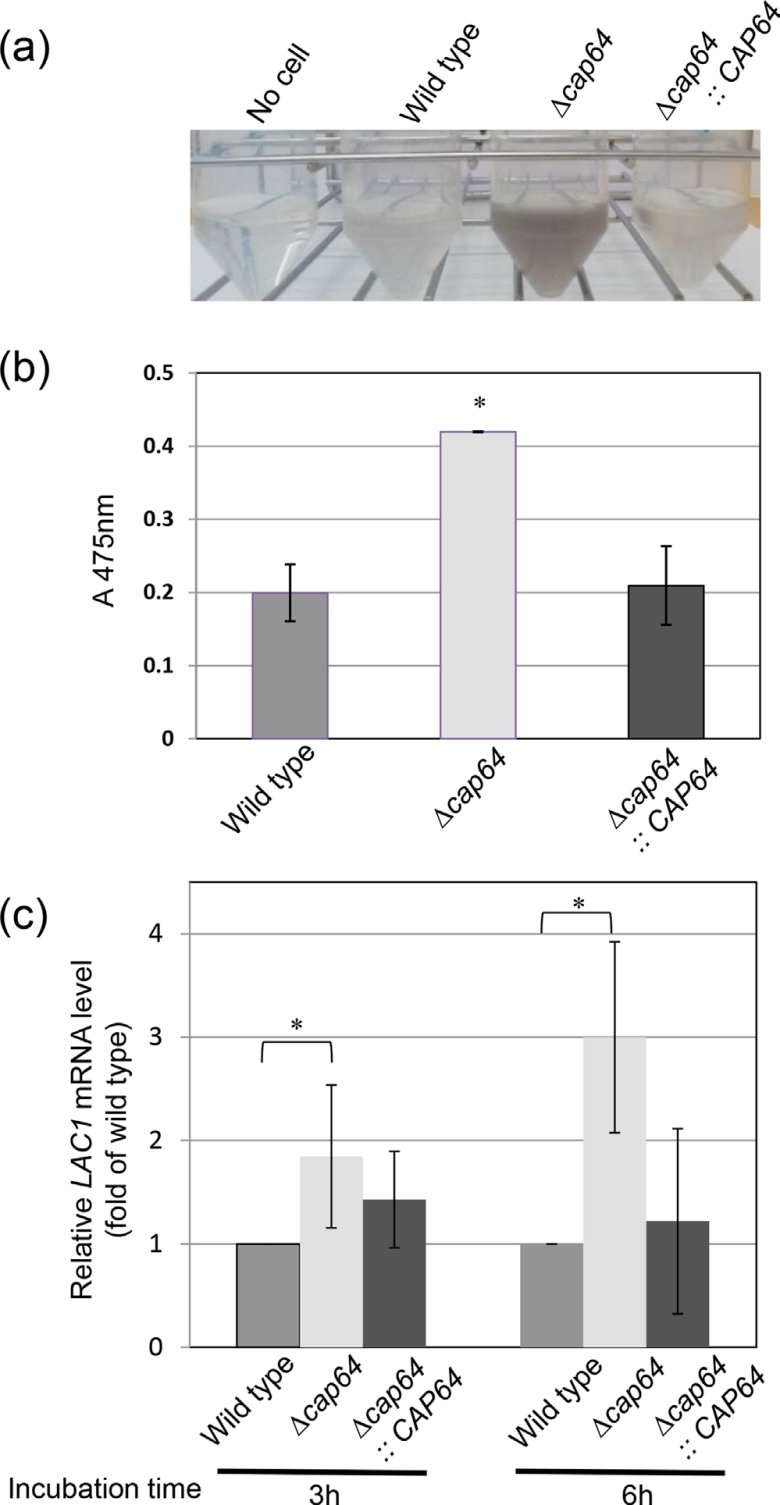
Deletion of the *CAP64* gene affects pigmentation in cryptococcal cultures. Pigmentation of *C. neoformans* strains was observed after incubating in asparagine medium for 24 h (**a**). The laccase activity of each strain was quantified by measuring the absorbance of pigments contained in the culture supernatant (**b**), and the transcription level of *LAC1*, which encodes laccase, was examined for each strain after culturing in asparagine medium for 3 or 6 h (**c**). Student’s *t*-test was used to compare them. Experiments were carried out in triplicate. Values are reported as the mean±SD. *Statistically significant by Student’s *t*-test (*P*<0.05).

The enzyme laccase is well known as one of the virulence factors of *C. neoformans* and is produced and released by the vesicle secretion system [23–25]. If *CAP64* is required for vesicle acidification, it might affect laccase secretion as well. Cells of Δ*cap64*, the wild-type, and the *CAP64* complement strains were incubated in asparagine medium to induce laccase production and the amount of pigment from each culture supernatant was determined. Fig. 4a shows the observation of pigmentation of the wild-type, Δ*cap64*, and complement strains after incubation for 24 h in asparagine medium. The colour of the Δ*cap64* culture supernatant became darker than the other two culture supernatants, suggesting that the Δ*cap64* cells produced more pigment. The absorbance of the Δ*cap64* culture supernatant was 0.420, which was 2.1-fold the absorbance of the wild-type culture after 24 h incubation in asparagine medium (Fig. 4b). Cells of the wild-type, Δ*cap64* and the *CAP64* complement strains were incubated in asparagine medium for 3 or 6 h, and the amount of *LAC1* transcript was analysed by qRT-PCR (Fig. 4c). The amounts of *LAC1* transcript in the Δ*cap64* cultures with 3 h and 6 h incubation were 1.85 or 3.00-fold those in the 3 h and 6 h wild-type cultures. The amounts of *LAC1* transcript in the *CAP64* complement cultures with 3 h or 6 h incubation were 1.43 or 1.22-fold the amounts in the corresponding wild-type cultures. The amount of *LAC1* transcript in the Δ*cap64* culture tended to increase, but there was no significant difference in the *LAC1* transcript between the Δ*cap64* strain and the *CAP64* complement strain.

### Effect of deletion of *CAP64* on *VHP1* or *SAV1* transcription

**Fig. 5.**
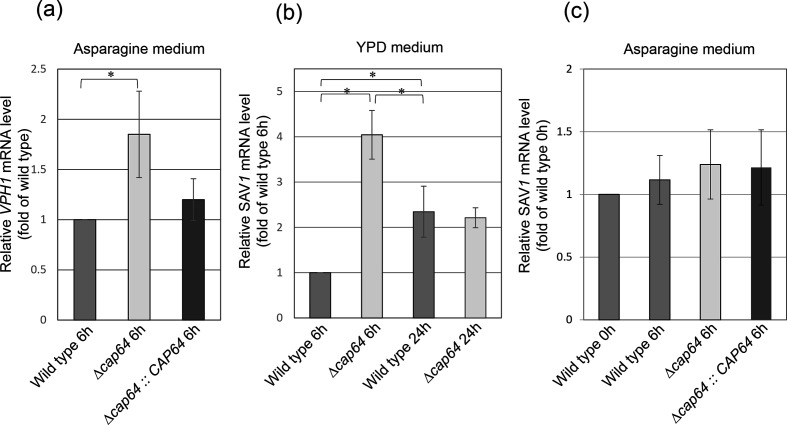
The amounts of *VPH1* or *SAV1* transcript in the wild-type and Δ*cap64* strain cultures were compared. The amount of *VPH1* transcript was examined after culturing in asparagine medium for 6 h (**a**), and the amount of *SAV1* transcript was examined after culturing in YPD medium for 6 or 24 h (**b**), and after culturing in asparagine medium for 6 h (**c**). Student’s *t*-test was used to compare them. Experiments were carried out in triplicate. Values are reported as the mean±SD. *Statistically significant by Student’s *t*-test (*P*<0.05).

Quinacrine accumulation is associated with the vesicular (H^+^)-ATPase protein pump gene *VPH1*, and thus deletion of the *CAP64* gene was expected to have some effect on the transcription of *VPH1*. We have already showed that the intracellular quinacrine accumulation was not observed in Δ*cap64*, but was clearly seen in the wild-type strain when cells were grown in YPD liquid medium [3]. The amounts of *VPH1* transcript in the wild-type, Δ*cap64,* and *CAP64* complement strain cultures were compared. Cells of the wild-type, Δ*cap64,* or *CAP64* complement strains were grown in YPD liquid medium for 6 h or 20 h, respectively, and the amount of *VPH1* transcript was analysed by qRT-PCR analysis. No signal was detected under this culture condition, although three independent experiments were performed. Next, cells of the wild-type, Δ*cap64,* or *CAP64* complement strains were incubated in asparagine medium for 6 h, and the amount of *VPH1* transcript was analysed by qRT-PCR analysis. This is because the amount of *LAC1* transcript in the culture with 6 h incubation was higher than that in the culture with 3 h incubation in the Δ*cap64* strain (Fig. 4c). Cells of the wild-type or Δ*cap64* strain were cultured in asparagine medium for 6 h, and the amount of *VPH1* transcript in the Δ*cap64* strain was compared to that in the wild-type strain. The *VPH1* transcript of the Δ*cap64* strain was 1.8 times that of the wild-type strain (Fig. 5a). The amount of *VPH1* transcript in the *cap64* strain tended to be higher compared to that of the wild-type, but there was no significant difference between the Δ*cap64* strain and the *CAP64* complement strain (p value=0.0503, Fig. 5a).

Next, the amount of the *SAV1* gene transcript, which is involved in vesicle exocytosis, was measured. The wild-type strain or Δ*cap64* strain was cultured in YPD liquid medium for 6 or 24 h, and the *SAV1* transcript levels were analysed by qRT-PCR. The amount of *SAV1* transcript was represented as the ratio of the amount of transcript in each culture to the amount of *SAV1* transcript in the 6 h culture of the wild-type strain. The amount of *SAV1* transcript in the 6 h culture of the Δ*cap64* strain was 4.0-fold the amount in the 6 h culture of the wild-type. The amounts of *SAV1* transcript in the 24 h culture of the wild-type strain and Δ*cap64* strain were 2.3-fold and 2.2-fold the amounts in the 6 h culture of the wild-type strain, and there was not a significant difference in the amount of *SAV1* transcript between the 24 h culture of the wild-type strain and the 24 h culture of the Δ*cap64* strain. The *SAV1* transcript levels in the cultures performed in asparagine medium were also analysed. The *SAV1* transcript levels of the wild-type strain and Δ*cap64* strain, which were incubated for 6 h in asparagine medium, were not significantly different (Fig. 5c).

### Intracellular localization of Cap64

Because deletion of the *CAP64* gene eradicated the quinacrine accumulation and the *VPH1* gene coding for the vacuolar (H^+^)-ATPase proton pump was not transcribed in the YPD culture, the Cap64 protein may be localized intracellularly and may be associated with acidic organelles which are stained with quinacrine. In order to examine this hypothesis, CAP64-mCherry overexpression strains were constructed. The CAP64-mCherry cassette was constructed and introduced into the Δ*cap64* strain. Two types of plasmids were constructed, in which mCherry was placed at the N- or C-terminals of *CAP64* (Fig. 1). From these plasmids, the CAP64-mCherry cassette was amplified by PCR and transformed into cells of Δ*cap64*.

**Fig. 6.**
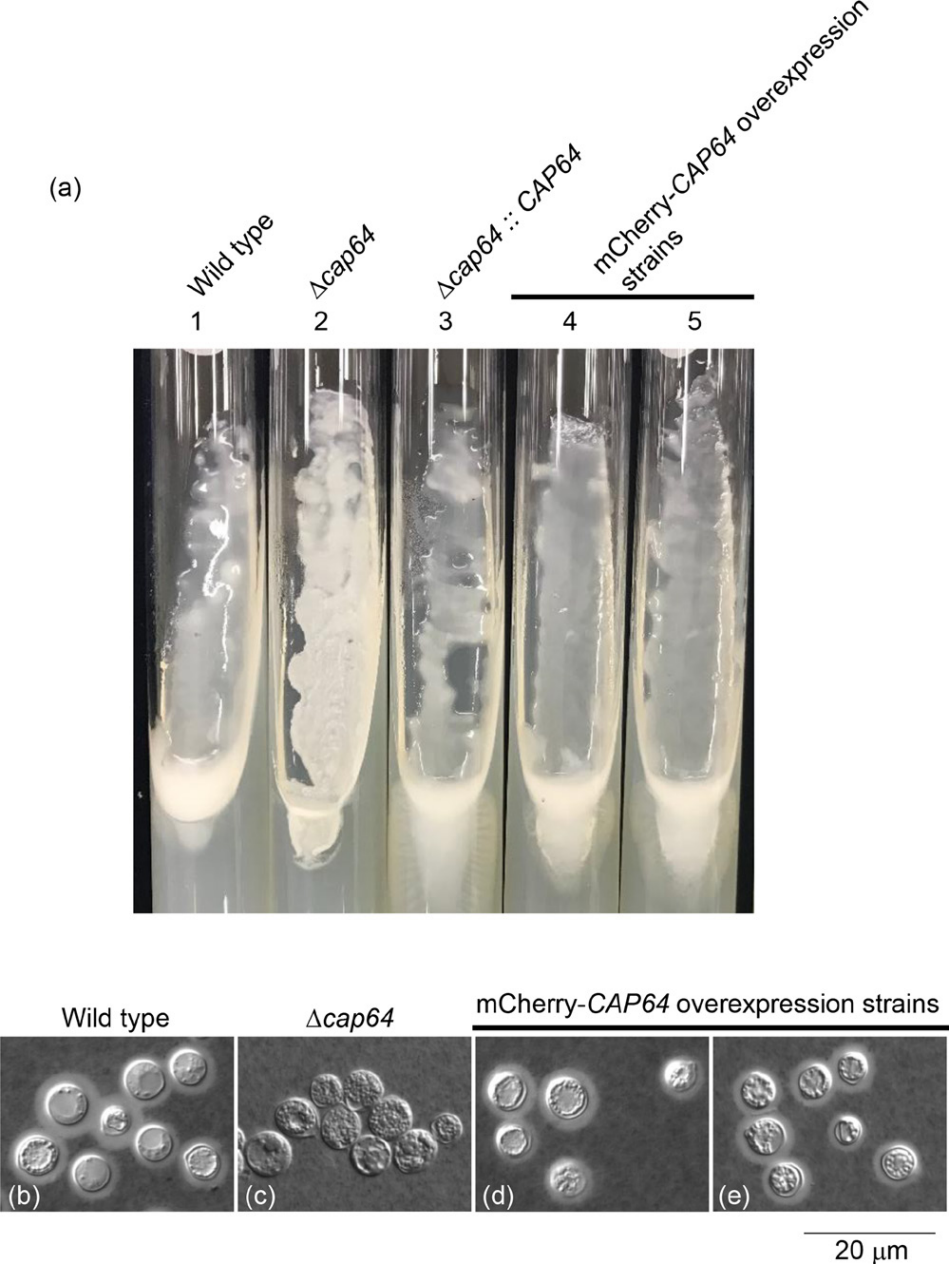
Observation of capsule formation of the CAP64-mCherry overexpression strains. The transformants of the CAP64-mCherry cassette amplified by PCR using pYI602 as a template were cultured on PDA slant medium for 1 week (**a**). Cells were observed after induction of capsule formation using a Leica microscope and DIC (b−e). The scale bar shows 20 µm.

When the Δ*cap64* strain was transformed with the CAP64-mCherry cassette obtained by PCR amplification using pYI601 as a template, ten transformants on YPD plates containing hygromycin B were obtained. Genomic DNA was isolated from each of these ten transformants and was confirmed to contain the DNA fragment of the CAP64-mCherry cassette by PCR amplification with the pro-cap64 and mcherry-R primers. However, these transformants were unable to produce a capsule (data not shown). Then, the Δ*cap64* strain was transformed with the CAP64-mCherry cassette obtained by PCR amplification using pYI602 as a template, and nine transformants on YPD plates containing hygromycin B were obtained. Among them, five transformants could produce a capsule (Fig. 6). The integration of the transformation cassette was confirmed by appropriate PCR followed by the sequence analysis. The amount of *CAP64* transcript of these four transformants was measured for the culture in YPD medium and was four-fold the amount of *CAP64* transcript in the wild-type strain. Therefore, these transformants were selected as CAP64-mCherry overexpression strains to analyse Cap64p subcellular localization.

**Fig. 7.**
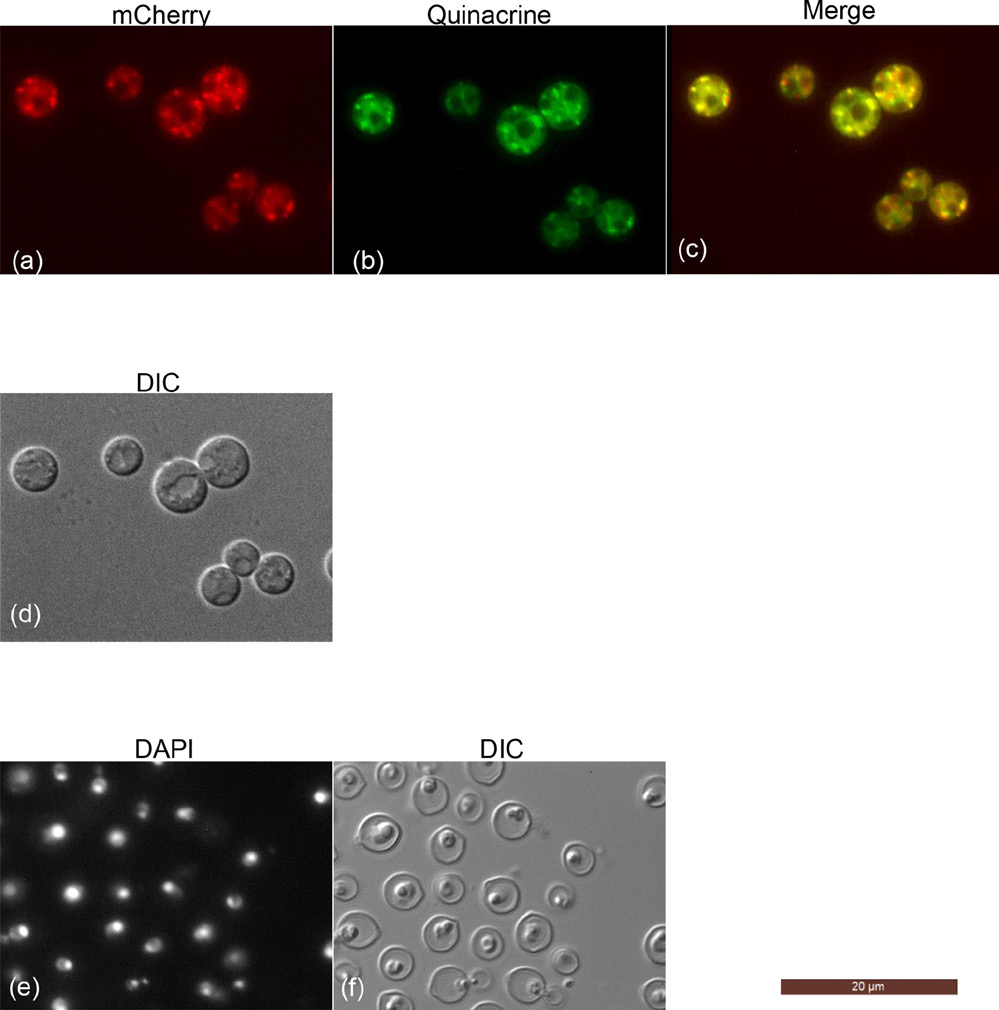
Cap64 is required for the vesicle acidification and is localized at the site of quinacrine accumulation. The Cap64 was tagged with mCherry at its N terminus and the resulting strains were cultured in YPD liquid medium at 25 °C for 20 h. The subcellular localization of mCherry of Cap64 (**a**), quinacrine accumulation (**b**), the merged image (**c**), and DAPI morphology (**d**) were visualized under a Leica microscope and DIC (d−f). The scale bar shows 20 µm.

The CAP64-mCherry overexpression strains were cultured in YPD liquid medium for 20 h, and the cells were collected and stained with quinacrine or DAPI, and the fluorescence of mCherry, quinacrine or DAPI was observed (Fig. 7). The mCherry fluorescence was clearly accumulated in cells in a patch-like manner, and the sites of quinacrine accumulation coincided with the location of the mCherry fluorescence patches (Fig. 7a−c). The fluorescent pattern of the mCherry was different from that of the DAPI staining (Fig. 7e), although cells stained with DAPI were fixed with ethanol, but it was determined that Cap64p did not accumulate in the nucleus. This observation suggests that Cap64p accumulates in vesicles during the cell growth stages.

## Discussion

In *C. neoformans*, the *CAP64* gene is well recognized to be involved in capsule synthesis because a null mutant, Δ*cap64*, lacks a capsule [12–14, 26]. On the other hand, we reported that cells of the Δ*cap64* strains stained negative for DBB staining [3]. Therefore, *CAP64* appears to have a different role other than capsule synthesis, and the phenotype of the Δ*cap64* strain was reanalysed. As the result of morphological observation, the Δ*cap64* strain was found not only to have abnormal quinacrine staining morphology, but also to have a morphology in which daughter cells could not be separated from parent cells. In *Saccharomyces cerevisiae,* this phenomenon of daughter cells being inseparable from parent cells is known to arise partly as the result of a failure in the extracellular secretion of mother/daughter separation enzymes (MDS proteins), which act to separate daughter cells from parent cells [27]. If *CAP64* is associated with organelle acidification and intracellular trafficking, *CAP64* deletion should affect these phenomena, leading to abnormalities in the expression of genes related to intracellular transport or the vesicle secretory system. When we compared the transcription levels of *VPH1* and *SAV1* among the wild-type, Δ*cap64,* and *CAP64* complement strains, the amount of *VPH1* or *SAV1* transcript in the Δ*cap64* strain was increased compared to that in the wild-type or *CAP64* complement strain. Quinacrine diffuses across membranes and accumulates in intracellular acidic compartments, but the amounts of *VPH1* transcripts in the wild-type, Δ*cap64,* and *CAP64* complement strains after YPD medium cultivation were undetectable, although quinacrine accumulation was observed in cells of the wild-type and *CAP64* complement strains (data not shown). This result suggests that the *VPH1* transcript might be restricted by the components of the YPD medium, and there might be other genes associated with organelle acidification in YPD culture. On the other hand, when cells were cultured in asparagine medium, the amount of *VPH1* transcript in the Δ*cap64* strain was higher than that in the wild-type strain, suggesting that *VPH1* is involved in vesicle acidification of the laccase secretion system. Ericsson *et al*. analysed the Δ*vph1* mutant of the *C. neoformans* H99 strain and found that disruption of *VPH1* resulted in defects in capsule formation and laccase and urease expression, but they did not find the *VPH1* disruption effect in cells under relatively stress-free environmental conditions, such as YPD, neutral pH, and 30 °C [8]. This result is consistent with our *VPH1* transcript analysis. The yeast may contain paralogs that cause acidification of vesicles, which are required for growth under stress-free conditions. We also revealed that *CAP64* was highly transcribed during *C. neoformans* growth in YPD medium and that Cap64p was associated with vesicle acidification at the growth phase. But *CAP64* disruption seemed not to affect the activity of laccase or urease [16].

In *S. cerevisiae*, Vph1p or Stv1p is included in the V-ATPase complex as the ‘a’ subunit, and the Stv1 protein (similar to Vph1p) is a paralog of Vph1p [28–31]. Vph1p is localized to the vacuoles, whereas Stv1p is present in the Golgi apparatus or other compartments, which are endosome compartments, and acts as a V-ATPase [29–31]. Interestingly, it is known that intracellular glucose depletion is involved in the dissociation of the V-ATPase complex [30]. In *C. neoformans*, the presence of an *STV1* gene was not found in a search of cryptococcal genomic databases [8]. When the CAP64-mCherry overexpressing strain was observed, Cap64p was accumulated in a patch-like manner and a quinacrine accumulation site was observed at the same location, although the cells were grown in YPD medium and Vph1p was not expressed. In addition, the patch size was smaller than the vacuole, which appeared to be the size of a vesicle. Cap64p seems to have a function similar to Stv1p, but there is no homology to a subunit of the vacuolar (H^+^)-ATPase complex from diverse organisms including *S. cerevisiae*, and therefore, *C. neoformans* might express an Stv1-protein paralog and this paralog that is localized to the vesicles and acts as a V-ATPase. It is thus necessary to investigate whether Cap64p is contained in vesicles and whether Cap64p is a component of the V-ATPase.

When the wild-type strain and Δ*cap64* strain were grown in YPD medium, the amount of *SAV1* transcript of the Δ*cap64* strain was 4.0-fold that of the wild-type strain, indicating that the *SAV1* was highly expressed in the Δ*cap64* strain. But when these strains were incubated in asparagine medium, there was no detectable difference in the *SAV1* expression level between the wild-type strain and the Δ*cap64* strain (Fig. 5), although the laccase activity in the Δ*cap64* strain was high (Fig. 4). This is because *SAV1* was not directly involved in laccase secretion, suggesting that there may be genes that perform the same function as *SAV1*.

**Fig. 8.**
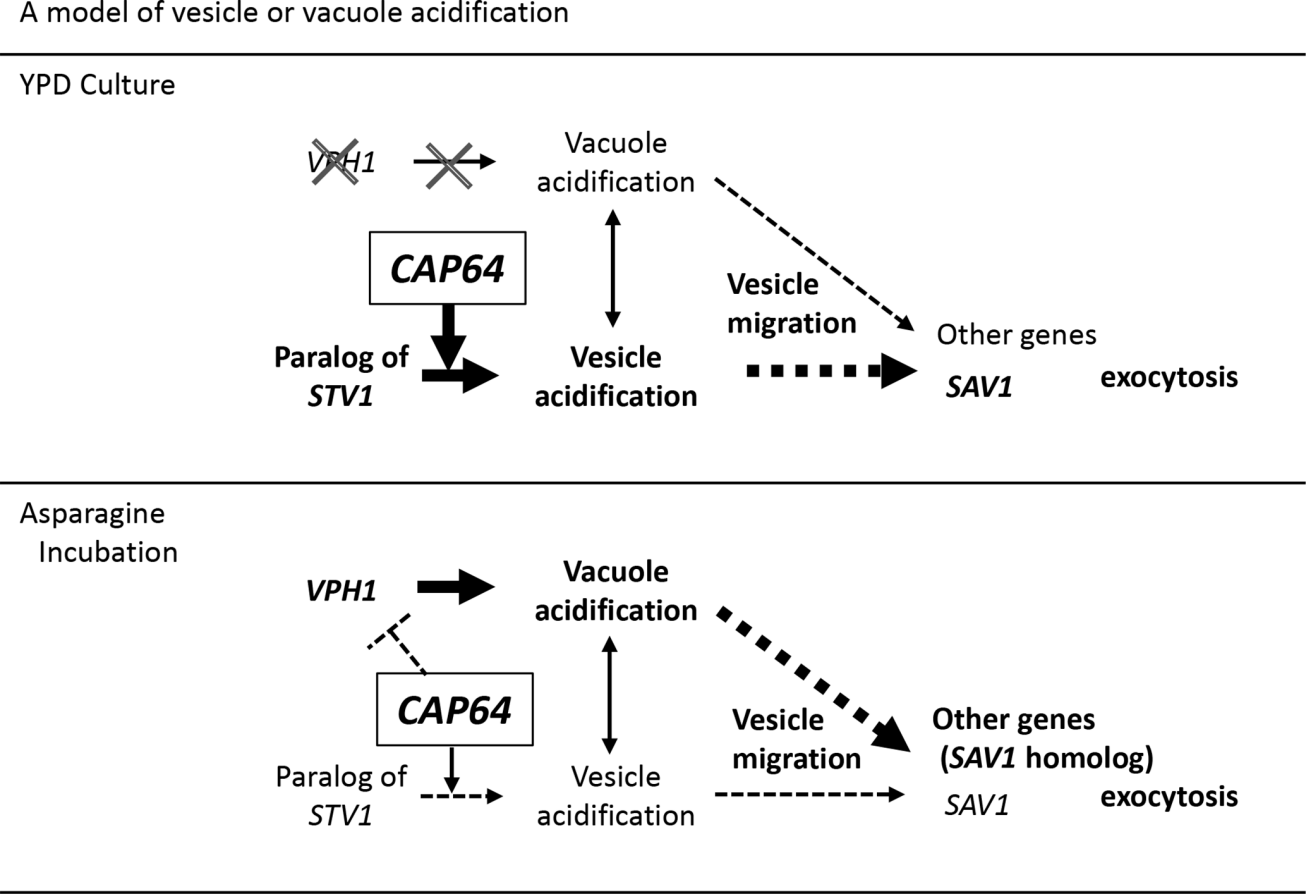
A model of vesicle or vacuole acidification. In YPD culture, *VPH1* is not transcribed and is not related with the formation of acidic vesicles during the cell growth. Therefore, a paralog of the *STV1* in *S. cerevisiae* may be present. *CAP64* is highly transcribed and seems to activate the paralog of the Stv1 protein, forming acidic vesicles. The acidification of vesicles regulates vesicle migration and membrane fusion, and the acidic vesicles containing some extracellularly secreted proteins fuse to the cell membrane via an action of *SAV1*. Some of the acidic vesicles fuse to the vacuole membrane to lyse unnecessary proteins. In asparagine medium, *VPH1* is highly transcribed and vacuoles are acidified. Because *VPH1* expression is up-regulated due to *CAP64* disruption, *CAP64* may suppress *VPH1* expression. Some acidified vacuoles might change to vesicles from which extracellular proteins such as laccase are secreted. Disruption of *CAP64* does not affect *SAV1* expression but activates laccase secretion. These findings suggest that the Sav1p homologue may be present and may be associated with laccase secretion [16].

Fig. 8 shows a model of vesicle or vacuole acidification. In YPD culture, *CAP64* activates the paralog of the Stv1 protein, forming acidic vesicles. The acidic vesicles contain some proteins secreted extracellularly which are necessary for the separation of mother and daughter cells. *VPH1* is not transcribed, but some acidified vesicles become vacuoles during the stationary phase of cell growth. When *CAP64* is disrupted, the vesicles are not acidified and vesicle migration is not regulated, leading to the high *SAV1* transcription. On the other hand, when cells are incubated in asparagine medium, *VPH1* is highly transcribed and the vacuoles are acidified. *CAP64* expression under this condition is reduced compared to that in YPD culture, and the reduction may be sufficient to suppress *VPH1* expression. When *CAP64* is disrupted, *VPH1* expression is up-regulated and some of the acidified vacuoles become vesicles that secrete extracellular proteins such as laccase. Disruption of *CAP64* does not seem to affect *SAV1* expression, suggesting that the Sav1p homologue may be present and may be associated with laccase secretion [16].

The DBB colony staining method is one of the methods used to distinguish basidiomycetous yeasts from ascomycetes [1, 2]. If *CAP64* is involved in the acidification of intracellular vesicles, it seems unlikely that *CAP64* plays a direct role in the ability of DBB colony staining to distinguish basidiomycetous yeasts from ascomycetous yeasts. However, many homologous genes of the Cas3p/Cap64p family have been isolated from basidiomycetes, and isolation of those genes is limited to the phylum Basidiomycota [32]. Basidiomycetous yeasts may have a genus-specific secretory system, and *CAP64* may be involved in it. On the other hand, we determined that the *PMT2* gene, which encodes *o*-mannosyl transferase, is also related with the DBB staining in *C. neoformans* [4]. The *PMT2* deletion mutant is negative by the DBB colony staining test but has a thick capsule around the cell wall [4]. Therefore, the DBB stained substrates may be *o*-mannosylated by Pmt2 at the ER and subsequently transferred to the Golgi apparatus and vesicles, and sorted to the cell membrane or cell wall. In a previous paper [3], it was shown that DBB stains the cell wall or cell membrane of the wild-type strain, so that the DBB-stained substrate is transported to the cell wall or cell membrane. In the *Δcap64* strain, the colonies are slightly stained by DBB compared to those of ascomycetous yeasts, but the cell wall and the cell membrane are not stained by DBB. This result suggests that the DBB-stained substrate is synthesized in the *Δcap64* strain, but it is not transported to the cell wall or cell membrane properly. It was also shown that in the *Δcap64* strain, quinacrine cannot accumulate in vacuoles as in the wild-type strain (Fig. 7f−g) [3]. In the present study, the location of the mCherry-tagged Cap64 protein was consistent with the location of intracellular quinacrine accumulation, as shown in Fig. 7. Since the vesicles are not sorted by *Δcap64*, it is considered that the DBB substrate could not be delivered to the cell wall or the cell membrane. If there are some proteins that complex with Cap64p and accumulate in vesicles, and these complexes maintain vesicle acidification, then these complex proteins should be detected and characterized, and their relationship with *PMT2* and *CAP64* should be analysed.
